# Opioid-associated modulation of respiratory-related cortical activity in dyspnoeic mechanically ventilated patients: An electroencephalographic study

**DOI:** 10.1016/j.aicoj.2025.100004

**Published:** 2026-01-16

**Authors:** Suela Demiri, Xavier Navarro-Suné, Marie-Cécile Niérat, Nicolas Wattiez, Maxens Decavèle, Côme Bureau, Sébastien Campion, Alexandre Demoule, Mario Chavez, Mathieu Raux, Thomas Similowski

**Affiliations:** a Sorbonne Université, INSERM, UMRS1158 Neurophysiologie Respiratoire Expérimentale et Clinique, F-75005 Paris, France; b AP-HP, Groupe Hospitalier Universitaire APHP-Sorbonne Université, Site Pitié-Salpêtrière, Département d’Anesthésie Réanimation, F-75013 Paris, France; c Sorbonne Université, INSERM UMR 1127, CNRS UMR 7225, Institut du Cerveau, Paris, France; d APHP, Groupe Hospitalier Universitaire APHP-Sorbonne Université, Hôpital Pitié-Salpêtrière, Service de Médecine Intensive et Réanimation, Département R3S, F-75013, Paris, France; e APHP, Groupe Hospitalier Universitaire APHP-Sorbonne Université, Hôpital Pitié-Salpêtrière, Département R3S, F-75013, Paris, France; f Fédération Hospitalo-Universitaire (FHU) "BREATH", APHP, Sorbonne Université, Inserm, Paris, France

**Keywords:** Dyspnoea, Respiratory-related brain suffering, Mechanical ventilation, Opioids, Electroencephalogram

## Abstract

**Background:**

Dyspnoea is frequent in mechanically ventilated patients and contributes to substantial distress and a heightened risk of post-traumatic stress disorder. It attests to respiratory-related brain suffering that must be actively managed. This is particularly challenging in noncommunicative patients, hence the interest of electroencephalographic surrogates. This study aims at characterizing the effects of opioids, proposed to relieve dyspnoea, on respiratory-related cortical activity in mechanically ventilated patients.

**Methods:**

In a 16-bed intensive care unit (ICU) over a 4-month period, we consecutively included eighteen mechanically ventilated patients with self-reported dyspnoea (visual analog scale –VAS–, communicative patients) or an observation-derived suspicion of respiratory-related brain suffering (noncommunicative patients, respiratory distress observation scores –RDOS–) persisting despite ventilator settings optimisation. Participants underwent electroencephalographic (EEG) recordings before and after administration of intravenous opioids. Respiratory-related cortical activity was assessed using covariance-based connectivity analysis, preinspiratory potentials (PIPs), and time-frequency analysis (TFA) time-locked to inspiration. To disentangle respiratory-specific from general effects of opioids, TFA was also computed from randomly selected EEG segments.

**Results:**

Opioids reduced dyspnoea evaluated by VAS and RDOS without significant sedation. EEG covariance analysis showed changes in brain state in 15 of 18 patients. PIP occurrence was variable and not modulated by opioids. TFA revealed statistically significant opioid-induced modulations in low beta and high beta power bands time-locked to inspiration, not observed in randomly timed analyses hence a specific effect of opioids on respiratory-related cortical networks.

**Conclusion:**

Opioids can relieve dyspnoea in mechanically ventilated ICU patients and modulate respiratory-related cortical activity beyond their general effects on cortical electrogenesis. This suggests that their effects on breathing control are not limited to brainstem mechanisms.

## Background

One third of invasively mechanically ventilated critically ill patients report dyspnoea during their intensive care unit (ICU) stay [1]. This symptom, which conveys an upsetting or distressing awareness of breathing [2], is one of the manifestations of respiratory-related brain suffering (a term that describes the abnormal brain reactions to disordered respiratory afferents [1]) together with changes in breathing pattern, autonomic stress and behavioral cues. It reflects intense distress and is associated with a heightened risk of developing post-traumatic stress disorder [1]. This makes its management a therapeutic priority.

Opioids have been extensively studied in patients with chronic dyspnoea that persists despite aetiological treatment [3], and are widely used to alleviate dyspnoea in palliative care at the end of life [4]. In contrast, their use as anti-dyspnoeic agents in acute care settings—including among mechanically ventilated patients—remains largely undocumented. Studying their effects and mechanisms of action in this context thus appears warranted.

Yet many mechanically ventilated patients cannot reliably communicate with their caregivers to signal dyspnoea or its relief after therapeutic interventions. To guide treatment in such cases, electroencephalographic (EEG) biomarkers of respiratory-related brain suffering have been explored to possibly serve as dyspnoea surrogates. Preinspiratory potentials (PIPs), analogous to the readiness potentials seen during movement preparation, have been associated with dyspnoea and shown to respond to various dyspnoea-relieving treatments [[5], [6], [7], [8]]. Riemannian continuous covariance analysis can also aid in identifying brain state changes in response to respiratory interventions [5,6,9,10]. Both approaches, which relate to cortical connectivity, have shown promise in a pilot study involving opioids prescribed to relieve dyspnoea in ventilated patients [5], but they are limited by certain methodological constraints. Riemannian analysis captures spatial changes in cortical connectivity in a rapid and sensitive manner, but does not specify the origin of the change: its respiratory nature can only be inferred from context. PIPs reflect respiratory-specific preparatory motor activity, but they require the analysis of numerous respiratory cycles and suffer from a low signal-to-noise ratio.

It is also possible to assess the influence of breathing on cortical electrogenesis through event-related time frequency analysis (TFA) [9]. TFA reveals frequency-specific modulations of electrocortical power time-locked to breathing, without the above limitations of Riemannian analysis and PIPs determination. Building on this method, we aimed to determine whether opioids could at the same time relieve dyspnoea in mechanically ventilated patients [5] and modulate respiratory-related cortical activity as captured with TFA –consistent with their known ability to depress respiratory-related cortical networks [11]. We therefore tested the hypothesis that opioids would alleviate clinical manifestations of respiratory-related brain suffering while altering the respiratory-related EEG TFA signature independently of their nonspecific effects on cortical electrogenesis [12,13].

## Methods

### Settings

The study was conducted over 4 months in a 16-bed intensive care unit (ICU) in Pitié Salpêtrière University Hospital, Paris, France. It was approved by the appropriate ethics committee (Comité de Protection des Personnes Ile de France VI). Informed consent was obtained from all the patients or their relatives.

### Study population

Patients were eligible for the study if they met the following criteria: (1) they had been mechanically ventilated via an endotracheal tube (invasive ventilation) for more than 24 h; (2) they had a score between −2 and +2 on the Richmond Agitation-Sedation Scale (RASS) [14]; (3) their breathing frequency exceeded 24. min^−1^ (an arbitraty threshold chosen to ensure an elevated neural drive to breathie) (4) they reported a dyspnoea rating of 4 or higher on a 10-cm visual analogue scale (VAS), with "no respiratory discomfort" on the left boundary and "worst imaginable respiratory discomfort" on the right boundary, this despite the ICU staff's adjustment of ventilator settings. For patients unable to communicate using the dyspnoea-VAS (noncommunicative patients), eligibility was determined by a score of 6 or higher on the Respiratory Distress Observation Scale (RDOS) [15] (Table S1, electronic supplement). Exclusion criteria were: age below 18, pregnancy, a known status epilepticus, hemodynamic instability, ongoing sedation within the last 24 h, or any condition that would preclude EEG recording.

### Collected variables

*Patients' description.* Age, body mass index (BMI), gender, Simplified Acute Physiology Score II (SAPS II), medical condition at admission, and past medical history were collected from medical records.

*Patients' status.* Awakening and interaction with the environment were evaluated using the RASS [14]. Consciousness and tolerance to the intensive care environment were assessed using the Adaptation to the Intensive Care Environment scale (ATICE) [16]. Pain levels were measured with the Critical Care Pain Observation Tool (CCPOT) [17].

*Dyspnoea and signs of respiratory-related brain suffering.* In communicative patients, dyspnoea was directly evaluated using a 10-cm visual analogue scale (VAS), as described above. In noncommunicative patients, respiratory discomfort was assessed using RDOS [15] and a modified RDOS excluding breathing frequency (to account for the specific effects of opioids on this variable). In the absence of a validated minimal clinically important difference for dyspnoea in mechanically ventilated ICU patients, a one-centimetre reduction on the 10 cm dyspnoea VAS was used as a threshold for clinical improvement, by analogy with MCIDs reported in chronic respiratory settings [18,19] and with a seminal study of dyspnoea in mechanically ventilated patients where the authors considered ventilator settings to be involved in the pathogenesis of dyspnoea when the post-intervention 10 cm VAS rating was at least 1 cm lower than the pre-intervention value [20]. As for RDOS, a one-point reduction was chosen as the threshold in view of palliative care data suggesting that this represents the difference between "severe dyspnea" and "moderate dyspnea" [21].

*Ventilatory variables.* Airway pressure was measured using a ±100-cm H_2_O linear differential pressure transducer (DP15−34, Validyne, Northridge, CA, USA) connected to the tracheal tube. Airflow was measured with a flow sensor (Flow Sensor 279331, Hamilton Medical AG, Rhazuns, Switzerland) placed in series with the tracheal tube and connected to a ±2-cm H_2_O differential pressure transducer (DP45−18, Validyne, Northridge, CA, USA). End-tidal carbon dioxide (ETCO_2_) was measured directly from the tracheal tube using a mainstream device (MicroStream®, Covidien, Dublin, Ireland). All signals were sampled at 40 Hz and recorded for subsequent analysis using Labchart 7.3® (ADInstruments, Dunedin, New Zealand). Tidal volume was calculated post-hoc by temporal integration of the airflow signal. Data analysis was performed offline using Matlab (The MathWorks, Natick, MA, USA). The onset of inspiration was defined from the flow signal (PowerLab 16/35, ADInstruments, Dunedin, New Zealand).

*Electromyographic recordings.* Electromyographic (EMG) signals were recorded using surface electrodes placed at the anatomical landmarks of the alae nasi, the fourth right intercostal muscle, and the right middle scalene muscle in the neck [22]. The signals were filtered using a 20-to-500-Hz passband and recorded at a 1 kHz sampling rate for offline analysis.

*EEG recordings.* Electroencephalographic (EEG) signals were recorded using a 32-surface electrode system (Acticap®, Brain Products GmbH, Germany) positioned according to the international 10−20 system (EasyCap®, Brain Products GmbH, Germany). Ground and reference electrodes were placed along the central midline. Electrode impedance was maintained below 5 kΩ across all experimental conditions using an electrode conducting gel (Lectron II®, Brain Products GmbH, Germany). EEG signals were amplified and recorded at a 256 Hz sampling rate (Recorder®, Brain Products GmbH, Gilching, Germany) for offline analysis. The pneumatic signal was integrated into the EEG recording (ExG Aux Box V2®, Brain Products GmbH, Gilching, Germany) to facilitate ventilatory-based EEG signal analysis. Additionally, head movement signals were captured using a 3D-acceleration sensor (3D-Acceleration Sensor®, Brain Products GmbH, Gilching, Germany) [23].

### Experimental protocol

All patients received pressure support (PS) mechanical ventilation using a Servo-I ventilator (Maquet Critical Care, Solna, Sweden). Prior to the study's initiation, endotracheal tube suctioning was performed, and ventilator settings were first adjusted to achieve a tidal volume (VT) of 6 ml·kg^−1^ if below this value. Patients were then evaluated using the dyspnoea VAS (for communicative patients) and/or RDOS (for all patients), with further ventilator adjustments made as needed, at the discretion of the attending physician. These could include increased trigger sensitivity, reduced positive end-expiratory pressure, increased inspiratory flow under assist-control ventilation, switching from assist-control to pressure support, or increasing the level of pressure support. Dyspnoea VAS and/or RDOS were then repeated to identify persistent dyspnoea. In its presence (according to inclusion criteria), the study then began with a first 30-minute signal acquisition period, referred to as the "initial persistent respiratory distress" condition, was then recorded. Following this, patients received titrated opioids to alleviate dyspnoea, administered according to ICU protocols and at the discretion of the attending physician, independent of the study. The opioid intravenous titration protocols were as follows:

- morphine: Initial dose of 0.05–0.1 mg kg^−1^, followed by 0.5 mg every 3 min;
- sufentanil: Initial dose of 0.05–0.1 μg kg^−1^, followed by 0.5 μg every minute;
- remifentanil: Continuous infusion at 0.05 μg kg^−1^·min^−1^.

After a 5-minute delay, chosen to exceed the known onset of action of the slowest-acting opioid used (typically within 3–4 min for morphine, 2 min for sufentanil, and 1 min for remifentanil), dyspnoea VAS and RDOS were repeatedly assessed until a one-point decrease in either VAS or RDOS was observed. Titration was then stopped, and a second 30-minute recording, referred to as the “opioid” condition, was conducted. Throughout both study conditions, ventilator settings and inspired oxygen fraction were kept constant.

### Signal processing and analysis

*EMG recordings.* The root mean square (RMS) of the raw signal from each muscle was calculated offline using Labchart 7.7 (ADInstruments, Dunedin, New Zealand). Ensemble averaging of the RMS for each condition was performed to identify the onset of phasic activity and to quantify the area under the curve [24]. The maximum activity of each muscle was determined offline by calculating the peak amplitude of the RMS (Peak Analysis, Labchart 7.7, ADInstruments, Dunedin, New Zealand).

*EEG processing 1: Continous covariance-based connectivity analysis.* The effects of opioid administration on cortical connectivity were assessed on an individual patient basis using a multichannel covariance-based classifier approach, as previously described [6,9,25]. This method involves comparing EEG recordings between a learning condition (here the "initial persistent respiratory distress" recording period) and a subsequent test condition (here the "opioids" recording period) to evaluate the classifier's ability to detect changes in the spatiotemporal EEG signature reflecting a brain state modification. To achieve this, the raw EEG signal from F3, Fz, F4, C3, Cz, C4, FP1 and FP2 was first segmented in 5-second sliding, 50% overlapping windows, and passband-filtered between 8 and 24 Hz for the purpose of enhancing mu rhythm as surrogate of motor cortical activity. Artefacts were then automatically rejected, based on identification of segments containing outlier values of amplitude, linear trend, joint probability or kurtosis. Rejection criterion was based on Z-score. Covariance matrices were then constructed to describe the EEG spatial dynamics. A one-class learning approach was used to define the reference prototype, made of 20 matrices (100 seconds) from the “initial persistent respiratory distress”. A total of 10 learning periods were repeated randomly to define the baseline. The statistical distance between any given EEG covariance extracted from the opioid condition and the initial persistent respiratory distress condition were plotted as a function of time. Distance was then compared using a threshold beyond which the EEG covariance became statistically different from the reference situation. For each patient, this threshold was based on the distribution of the distances between all the covariance matrices estimated from the reference period where no significant changes were expected. Finally, classification performance was evaluated using a 10-fold cross-validation process, where the first period was divided into 10 equal EEG segments. Comparisons between nine of these segments randomly selected and the data from the second period were repeated nine times, accounting for all possible combinations. The results were represented as Receiver Operating Characteristic (ROC) curves [26], with prediction areas under the curve (AUCs) ranging from 1 (perfect discrimination) to 0.5 (random discrimination). An AUC ≥ 0.7 was considered satisfactory for identifying cortical activity modifications [6,27] (Figure S1).

*EEG processing 2: preinspiratory potentials.* To examine the presence of PIPs, also on an individual patient basis we used a previously described methodology [6,7,28]. Briefly, this approach involved averaging EEG segments time-locked to the onset of inspiration and examining them for a pre-inspiratory negativity occurring 0.5–1.5 seconds before inspiration. To achieve this, the EEG signal was preprocessed by referencing to earlobe electrodes, and applying a 0.05–10 Hz passband filter, chosen to balance signal preservation and noise suppression in the high frequency range, even at the cost of some sensitivity loss.The signal was then segmented into epochs time-locked to the onset of inspiration, starting 2500 ms before and ending 500 ms after. The criteria for EEG epoch rejection were based on the following statistical thresholds: (i) voltage amplitude: epochs were marked for rejection if the signal amplitude exceeded ±50 μV, primarily targeting large, non-physiological events (e.g., electrode movements or muscle bursts); (ii) standard deviation: epochs were flagged if any channel’s activity exceeded 5 standard deviations (SD) from the mean (indicating excessive localised noise); (iii) Kurtosis: epochs were rejected if their kurtosis deviated by more than 5 SD from the mean (helping to identify sharp, non-Gaussian transients, often muscle-related). All flagged epochs were then visually inspected to ensure that rejections were appropriate and not overly conservative, preserving as much usable events as possible. This process led to rejecting 16.4 ± 5.3% of segments in the “initial persistent respiratory distress” condition, compared to 16.6 ± 6.0% in the “opioid administration” condition. The remaining artifact-free epochs were then ensemble averaged. The presence of a slow negative shift preceding inspiration onset (the PIP) in the averaged EEG traces (either Cz or FCz) was assessed in a blinded manner by three independent researchers with expertise in the field (MR, SD, M-CN).

*EEG processing 3: Time frequency analysis.* Finally, we performed a time-frequency analysis (TFA) to assess respiratory-locked cortical activity. EEG signals from the Cz electrode were segmented into epochs spanning −1.5 to 0.5 s relative to inspiratory onset (0 s), with a baseline period defined from −1.5 to −1.2 s for relative power normalization [9]. For each segment, time-frequency maps (TFMs) were computed using Morlet wavelet convolution, providing spectral power estimates across frequencies (y-axis) and time (x-axis). Power values were expressed as relative changes compared to the baseline interval. These single-subject TFMs were trial-averaged for each patient, and a grand-average TFM was computed across all 18 patients to examine population-level respiratory-locked EEG dynamics. To disentangle the influence of opioids [[29], [30], [31], [32]] and respiratory-related EEG power spectrum [9] we implemented the following procedure. First, we time-locked the TFA to the onset of inspiration, using a method similar to that employed for PIP identification (TFA_VENTIL_, expected to be sensitive to any change in cortical electrogenesis related to ventilation). Next, we performed the TFA on EEG segments of identical duration, but randomly selected within the same condition, irrespective of ventilatory events (TFA_RANDOM_, expected to be sensitive to any change in cortical electrogenesis apart from those related to ventilation, *ie* sensitive to the effects of opioids). This analysis was conducted during both the initial persistent respiratory distress condition and the opioid condition. Comparing TFA_VENTIL_ across conditions enabled the detection of opioid-related changes in electrocortical activity, whether intrinsic or respiratory-related. Comparing TFA_RANDOM_ across conditions allowed for the identification of opioid-related changes in electrocortical activity independent of any respiratory influence. Finally, comparing TFA_VENTIL_ to TFA_RANDOM_ during the opioid administration condition enabled the identification of a specific opioid effect on respiratory-related EEG activity.

### Statistical analysis

Due to the exploratory nature of the study, no reliable sample size calculations could be performed, and the study utilised a convenience sample of consecutive patients.

*Inferential statistics.* The normality of data distribution was assessed with Kolmogorov-Smirnov test. Continuous variables are summarised as means (with standard deviations) for normally distributed data or medians (with interquartile ranges) for non-normally distributed data. Categorical variables are presented as n (percentage). A Wilcoxon log-rank test was used to compare continuous variables, while McNemar's test with continuity correction was applied to assess changes in the occurrence of PIPs. The AUC for EEG analyses was identified as the value approaching 1. Statistical analyses were performed using SPSS version 21.0 (IBM Corporation, Armonk, NY, USA). A probability of a type I error below 0.05 was considered statistically significant.

*EEG statistical processing.* To determine significant spectral perturbations, we used nonparametric permutation testing with a two-dimensional clustering approach [33], correcting for multiple comparisons across the time-frequency plane. The between-condition comparisons were performed using independent-samples *t*-tests, which define clusters of adjacent time-frequency points exceeding a p < 0.05 threshold. 1000 random permutations were performed to estimate the null distribution (no difference between conditions) of cluster masses. Clusters in the observed data were deemed significant if they exceeded the 95th percentile of the permuted null distribution (*α* = 0.05). Statistically significant clusters were visualised as grayscale-shaded regions on TFMs, distinguishing respiratory and opioid-related effects.

## Results

### Population

Eighteen patients were consecutively included in the study, of whom ten were female (56%). Their demographic and clinical characteristics are summarised in Table 1 together with the reasons for their ICU admission. Seventeen patients were connected to the ventilator via a cuffed endotracheal tube, while one was ventilated through a tracheostomy tube. Seventeen patients were ventilated in pressure support ventilation (PS) mode, and one patient was managed with assist-control ventilation (ACV), a volume-controlled ventilation with spontaneous breathing mode. Seventeen patients had been weaned from continous opioids infusion at least 24 hours before study enrollment, while one patient still received continuous intravenous sufentanil at a dose of 0.004–0.006 μg kg^−1^·h^−1^ (this dose was considered sufficiently low to allow inclusion; the sulfentanil protocol was used in this patient, see above).

**Table 1:** Characteristics of the study participants.

Variable	Value
**Age** (*years*)	63 [56; 68]
**Body Mass Index** (*kg.m^−2^*)	26 [22; 30]
**SAPS II**	40 [35; 51]
**Reasons for initiating mechanical ventilation**	
*De novo* acute respiratory failure	6 (33%)
Acute on chronic respiratory failure	2 (11%)
Sepsis	8 (45%)
Other	2 (11%)
**Mechanical ventilation duration at inclusion (days)**	5 [3; 7]
**Gas exchange***(on inclusion)*	
pH	7.44 [7.37; 7.45]
PaCO_2_ (*mmHg*)	37 [33; 44]
PaO_2_/FiO_2_ (*mmHg*)	230 [155; 226]
HCO_3_^−^ (*mmol.l*^−^*^1^*)	25 [20; 29]
**Dyspnoea assessment***(on inclusion)*	
VAS (c*m*)	6 [5; 7]
RDOS	8 [7; 9]
IC-RDOS	6.3 [5.2; 6.7]
**ATICE***(on inclusion)*	13 [11; 15]
**RASS***(on inclusion)*	0 [-1; 0]
**CPOT***(on inclusion)*	4 [3; 5]
**ICU length of stay** (*days*)	12 [6; 26]

**SAPS II** Simplified Acute Physiological Score 2; **P_a_CO_2_** arterial carbon dioxide partial pressure; **P_a_O_2_** arterial oxygen partial pressure; **FiO_2_** inspired oxygen partial fraction; **HCO_3_**^−^ arterial bicarbonates; **VAS** Visual Analogic Scale for respiratory discomfort; **RDOS** Respiratory Distress Observation Scale, **ATICE** Adaptation to the Intensive Care Environment, **RASS** Richmond Agitation Sedation Score, **CPOT** Critical Care Pain Observation Tool.

Data are shown as median [IQR] or values (percentages).

### Opioid administration and clinical results

Eight patients received morphine (from 0.10 to 0.22 mg kg^−1^, median 0.12 [Q1 0.11; Q3 0.15]), eight received sufentanil (from 0.13 to 0.37 μg kg^−1^, median 0.20 [Q1 0.15; Q3 0.22]), and two received remifentanil (0.05 mg kg^−1^·min^−1^ and 0.10 mg kg^−1^·min^−1^). Opioid administration reduced respiratory discomfort, as demonstrated by a decrease in dyspnoea-VAS ratings from 6 [5; 7] to 5 [4; 5] (p = 0.02) among nine communicative patients, and a reduction in the RDOS score from 8 [7; 9] to 6 [4; 7] in nine non-communicative patients (p = 0.03)(RDOS reduction in the 18 patients from 7.5 [7; 8.75] to 5.5 [3.5; 7], p = 0.03). The difference in RDOS in non-communicative patients remained significant after neutralizing the respiratory rate component in the score calculation (from 6 [6; 8] to 5 [3; 6], p = 0.01). Opioid administration also decreased the CCPOT score from 4 [3; 5] to 3 [2; 4] (p = 0.01) compared to baseline conditions. The ATICE score remained unchanged (13 [11; 15] both before and after opioid administration; p = 0.89), as did the RASS score (−1 [−1; 0] both before and after; p = 1; 5 patients RASS -2 before opioids (without change after), no patient RASS + 2). Opioids significantly altered the ventilatory pattern, increasing inspiratory time and decreasing respiratory rate (Table 2). Heart rate and oxygen saturation remained stable, while mean arterial pressure showed a significant reduction. No opioid-related side effects were observed. Of note, the one-point reduction in dyspnoea VAS or RDOS was not reached in 2 out of the 18 patients, in which cases the physician in charge chose to deepen sedation.

**Table 2:** Respiratory and haemodynamic data.

Variables	Initial persistent respiratory distress condition	Opioid condition	p
T_I_*(s)*	0.8 [0.7; 0.9]	0.9 [0.8; 1.4]	**0.001**
T_I_/T_TOT_	39 [35; 41]	37 [33; 39]	0.07
RR *(.min*^−^*^1^)*	29 [26;33]	24 [21; 31]	**0.03**
Ventilation*(l.min*^−^*^1^)*	12 [11; 14]	11 [9; 12]	0.89
Tidal volume *(ml)*	432 [387; 472]	444 [392; 478]	0.94
Tidal volume *(ml.kg*^−^*^1^)*	6.1 [4.3; 6.8]	5.7 [4.0; 6.8]	0.78
E_T_CO_2_*(mmHg)*	30 [25; 35]	31 [27; 39]	0.08
S_P_O_2_*(%)*	97 [95;98]	96 [93; 97]	0.06
HR *(.min*^−^*^1^)*	92 [82;104]	89 [82; 104]	0.21
MAP *(mmHg)*	86 [74;94]	77 [66; 89]	**0.001**

T_I_ inspiratory time, T_I_/T_TOT_ fraction of inspiratory time over total cycle time T_TOT_, RR respiratory rate, E_T_CO_2_ end tidal carbon dioxide, S_P_O_2_ pulse transcutaneous oxygen saturation, HR heart rate, MAP mean arterial pressure.

Data are expressed as median and interquartile range.

### EMG recordings

Opioids did not influence accessory respiratory muscle activity. The maximal amplitudes and root mean square values of the electromyogram remained consistent across all conditions and respiratory muscles assessed (alae nasi, parasternal, and middle scalene) (Table 3).

**Table 3:** Respiratory muscles activity during initial persistent respiratory distressand following opioid administration.

Muscle activity	Initial persistent respiratory distress period	Opioids period	p
Scalene muscle maximum EMG (*mV*)	0.4 [0.1−1.3]	0.4 [0.1−1.2]	0.9
Scalene muscle EMG AUC (*mV^2^*)	0.1 [0.1−0.3]	0.1 [0.1−0.6]	0.5
Parasternal muscle maximum EMG (*mV*)	8.2 [4.1−20]	5.3 [2.6−19.4]	0.6
Parasternal muscle EMG AUC (*mV^2^*)	0.6 [0.1−2.1]	0.7 [0.1−1.8]	0.9
Alae nasi EMG maximum (*mV*)	3.8 [1.8−7.3]	2.8 [1.3−5.8]	0.4
Alae nasi EMG AUC (*mV^2^*)	0.6 [0.2−2.3]	0.4 [0.1−1.4]	0.3

EMG Electromyogram. AUC Area under the Curve.

Data are expressed as median and interquartile range.

### EEG recordings 1: continuous covariance-based connectivity analysis

**Fig. 1:**
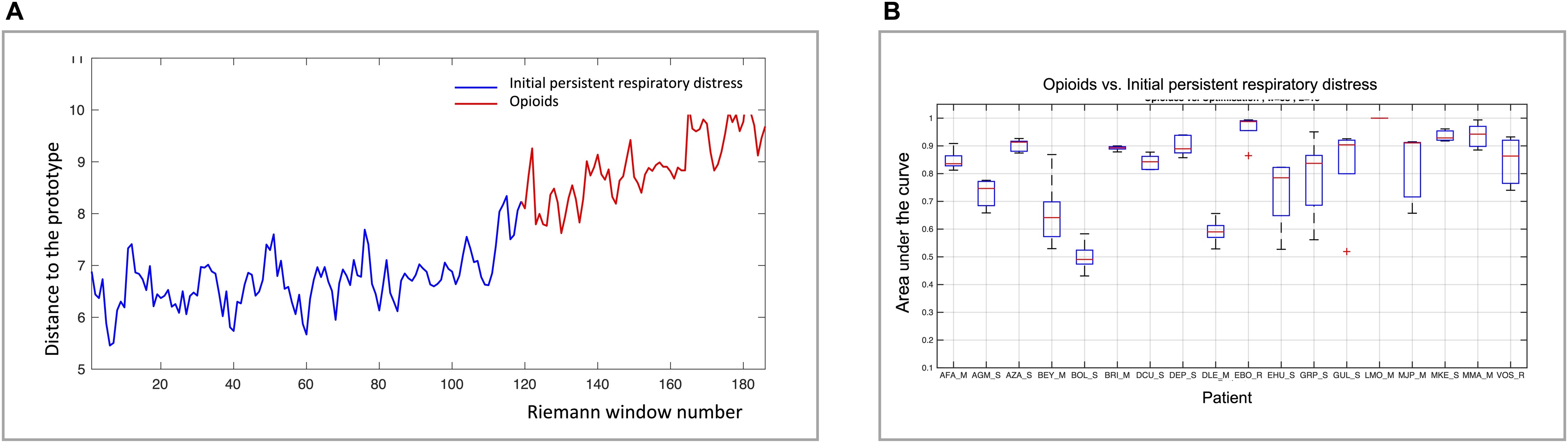
**Panel A**: representative example, from a single patient, illustrating the evolution of the statistical distance to the prototypic covariance matrix between the initial period of persistent respiratory distress (blue) and the opioid administration period (red). **Panel B**: Patient-by-patient results of the permutation process used to determine between state discrimination area under the curve according to the continuous covariance analysis (see Methods for details). The boxes represent the first quartile-third quartile range with indication of the median, the whiskers represent the minimum-maximum range.

The covariance-based analysis revealed changes in the EEG signal following opioid administration (Fig. 1A). Using the "initial persistent respiratory distress" condition as a reference, the area under the curve (AUC) for detecting changes in the EEG signal after opioid administration ranged from 0.46 to 1 (median 0.75, Q1 0.70, Q2 0.93), with 15 patients exhibiting an AUC of 0.7 or higher (which was considered the threshold for satisfactory identification of brain state changes)(Fig. 1B). The 3 patients in whom the AUC was below 0.7 were "non-responders" to opioids in terms of VAS and RDOS.

### EEG recordings 2: Pre inspiratory potentials

Four patients exhibited PIPs during the initial respiratory distress condition. In three of these cases, the administration of opioids was associated with the resolution of PIPs. Conversely, PIPs emerged following opioid administration in 4 of the 14 patients who had no PIPs after ventilator adjustments. Consequently, the incidence of PIPs in the study population was not significantly influenced by opioid administration (McNemar’s χ^2^ = 0.14, p = 1).

### EEG recordings 3: Time frequency analysis

**Fig. 2:**
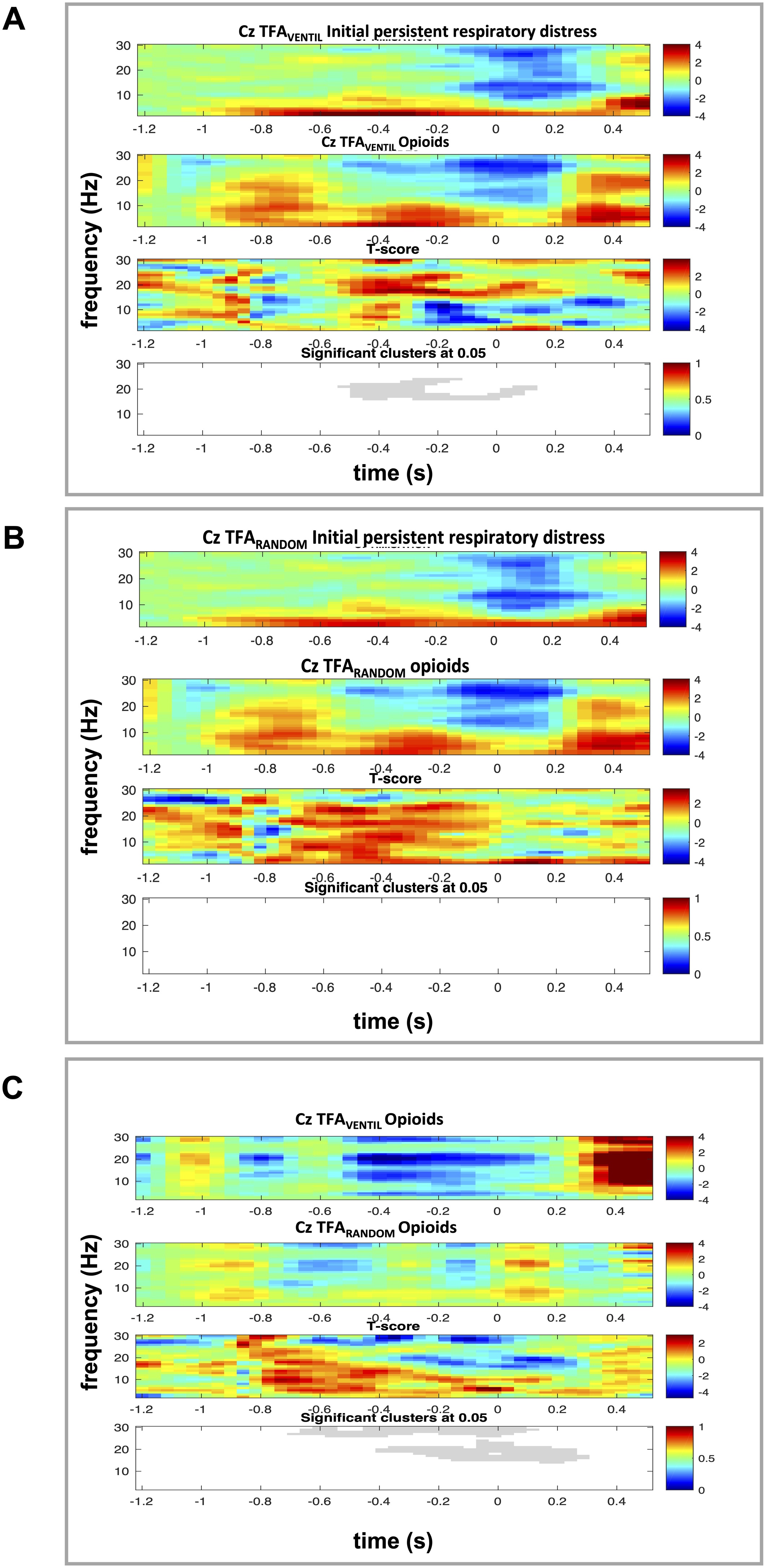
**Population averaged Time Frequency Analysis (TFA).** In all three panels, time is represented on the X-axis, with zero marking the onset of inspiration, and frequencies are shown on the Y-axis. Red indicates significant increases in EEG power, while blue indicates significant decreases. **Panel A:** TFA calculated from EEG segments time-locked with inspiration (TFA_VENTIL_). The first row presents the TFA results for the initial respiratory distress condition. The second row shows the TFA results for the opioid administration condition. In the first two rows, the color code represents the statistical comparison of EEG changes relative to the baseline. The third row contrasts the two preceding conditions. The fourth row displays statistically significant differences between conditions, represented in grayscale. **Panel B:** TFA calculated from randomly selected EEG segments (TFA_RANDOM_). The first row presents the TFA results for the initial persistent respiratory distress condition. The second row shows the TFA results for the opioid administration condition. In the first two rows, the color code represents the statistical comparison of EEG changes relative to the baseline. The third row contrasts the two preceding conditions. The fourth row displays statistically significant differences between conditions, absent in this case. **Panel C:** Comparison of TFA_VENTIL_ (first row) with TFA_RANDOM_ (second row) during the opioid administration condition. The third row contrasts the two preceding ones. The fourth row displays statistically significant differences between conditions. represented in grayscale.

Comparison of TFA_VENTIL_ between conditions revealed a statistically significant preinspiratory decrease in beta power, followed by a statistically significant postinspiratory increase in the same frequency range following opioid administration. (p < 0.05, Fig. 2A).

Conversely, the comparison of TFA_RANDOM_ during the initial respiratory distress condition with TFA_RANDOM_ during the opioids condition did not identify any change in the EEG power spectrum (Fig. 2B). Lastly, contrasting TFA_VENTIL_ with TFA_RANDOM_ during the opioid administration condition revealed statistically significant increases in preinspiratory low beta power and high beta power, followed by statistically significant increases in the same frequency ranges (Fig. 2C). These results indicate a specific effect of opioids on the respiratory-related network activated concurrently with respiratory discomfort, irrespective of the direct general effects of opioids on electrocortical activity.

## Discussion

### Summary of findings

In this study, opioid administration alleviated persistent dyspnoea or respiratory-related brain suffering in intubated mechanically ventilated ICU patients, with both communicative and noncommunicative individuals exhibiting significant reductions in dyspnoea or respiratory distress observation scores. Concomitantly, continuous EEG covariance analysis demonstrated opioid-induced brain state modifications, that could be attributed using time-frequency analysis (TFA) to a specific effect of opioids on respiratory-related cortical networks—manifested as modulations in low beta and high beta power bands—rather than to a generalised alteration of cortical electrogenesis.

### Data contextualisation

In our patients, opioid administration produced the expected clinical and physiological effects, including a reduction in CCPOT scores, a slight decrease in mean arterial blood pressure, a slowing of respiratory rate, and an increase in inspiratory time relative to the respiratory period [5,34]. These observations suggest appropriate dosing. Opioid administration also alleviated dyspnoea and/or reduced various RDOS values. Opioids have long been used to manage chronic dyspnoea in end-of-life care [35] and for extended periods in non-terminal patients with severe respiratory diseases [36], including chronic obstructive pulmonary disease [37,38]. There are no data to support their use in managing acute dyspnoea, except in the context of dyspnoea crises in terminally ill patients, where opioid prescription is accompanied by the acceptance of the principle of double effect [39]. The use of opioids to alleviate respiratory discomfort in mechanically ventilated patients or non-intubated acutely ill patients with respiratory failure, without any therapeutic limitation or end-of-life context, has been investigated in only two previous studies [5,40].

### Mechanisms of opioid-associated dyspnoea relief

A primary mechanism for reducing the intensity of dyspnoea involves decreasing the neural drive to breathe [2]. Opioids exert this effect at the brainstem level by acting as agonists on mu receptors in the pre-Bötzinger complex [see review inRefs. 41,42].The reduction in breathing frequency observed in our patients indicates that this mechanism contributed to the clinical relief described in our patients. However, the absence of significant changes in inspiratory muscle electromyograms suggests that the reduction in respiratory drive was not profound. A second mechanism for relieving dyspnoea involves modulating the brain's processing of respiratory-related afferents [2]. This has been well documented in mechanically ventilated ICU patients through interventions such as music listening or trigeminal stimulation [43]. Our EEG data suggest that opioids may have acted through this pathway in our patients. Furthermore, our findings indicate that the observed effects are not solely attributable to the known influence of opioids on cortical electrogenesis [44]. Indeed, in the present patient population, we did not observe opioid-related changes in global EEG activity (TFA_RANDOM_ comparisons). More importantly, the frequency power changes we observed were clearly present during the opioid condition when comparing TFAs locked to inspiration with randomly generated TFAs. This indicates a specific effect of opioids on the respiratory-related network activated concurrently with respiratory discomfort. This observation aligns with findings by Pattinson et al. [11], who, using functional magnetic resonance imaging in healthy individuals, showed that opioids profoundly reduced the breath-holding related urge to breathe, concomitant with decreased activity in the bilateral insula, frontal operculum, and secondary somatosensory cortex—a network also implicated in inspiratory loading-related dyspnoea [45]. Notably, activity in regions mediating motor control remained unaffected in that study [11]. This may explain the lack of consistent effects of opioids on PIPs in our patients, as PIPs typically reflect motor area involvement in dyspnoea pathogenesis. Yet since ventilator settings were adjusted prior to opioid administration in our study, patients were less likely to exhibit PIPs [6,7]. Additionally, polypnoea complicates the interpretation of PIPs, no data are available to guide this interpretation across vigilance states, and the presence of PIPs only partially overlap with that of dyspnoea [6].

### Methodological considerations

While promising, our study has some limitations, foremost among them a population size limited to 18 patients for pragmatic and convenience reasons. EEG acquisition in the ICU presents specific challenges, with multiple potential sources of interference related to both the environment and the patient’s condition. We addressed this through careful recording procedures and rigorous signal preprocessing, but this remains an important consideration, particularly in view of broader clinical applications. While the heterogeneity of opioids used broadens the applicability of the findings beyond a specific drug, the small population size precludes a comparative analysis of the differential effects of various opioids—particularly since remifentanil was used in only two cases. The inclusion of both communicative and noncommunicative patients necessitated the use of two distinct approaches to assess respiratory discomfort, introducing a potential source of bias. Here again, the limited sample size prevented a meaningful comparison between these two categories of participants. Additionally, two patients did not exhibit any reduction in dyspnoea-VAS or RDOS following opioid administration, making the data set used for ensemble averaging heterogeneous. If anything, this should have attenuated the observed effects. Finally, we cannot exclude that patient fatigue or fluctuations in attention, particularly over the course of the protocol, may have influenced responses independently of pharmacological effects. Despite these limitations, our study also has notable strengths. As mentioned earlier, it is among the few studies addressing respiratory-related suffering in acutely ill, mechanically ventilated patients, and the first to investigate the combined clinical and EEG effects of opioids in this context. Its design is particularly relevant to the study of dyspnoea that persists despite appropriate etiopathogenic measures (e.g., ventilatory underassistance correction). The multimodal EEG approach, including time-frequency analysis (TFA), which had not previously been employed for this purpose, contributes to advancing the understanding of cortical connectivity changes in this specific context.

## Conclusions

This study confirms that opioid administration can effectively relieve persistent dyspnoea in invasively mechanically ventilated ICU patients, as previously reported by Decavèle et al. [5]. Importantly, our findings provide mechanistic evidence that the observed effect is not solely mediated by a reduction in the neural drive to breathe at the brainstem level but also involves a cortical component. Evidence supports a specific action on respiratory-related cortical networks implicated in dyspnoea perception, rather than a generalised effect of opioids on cortical electrogenesis. These findings advocate for a carefully considered broader integration of opioids into therapeutic strategies aimed at managing persistent respiratory discomfort in critically ill patients under mechanical ventilation. This approach may be particularly valuable in addressing the clinical dilemma of balancing low tidal volumes for lung protection with higher tidal volumes for dyspnoea relief. However, any such use must take into account the other known risks of opioids, such as opioid-induced hyperalgesia, tolerance, drug interactions, etc. In particular, while bearing in mind that the adverse effects of opioids on ventilatory drive in patients with acute respiratory failure [40] are less likely to be of concern under mechanical ventilation, whether opioids can reliably alleviate dyspnoea without interfering with ventilator weaning remains an open and clinically critical question. We propose that our observations contribute to the rationale for further research needed to establish the optimal use of titrated opioids to relieve dyspnoea in this context –without oversedation–, particularly regarding patient selection, dosing regimens, duration of effet, and long-term safety.

## CRediT authorship contribution statement

Conceptualization: SD, MR, AD, TS.

Methodology: SD, MR, AD, TS, XNS, MC.

Investigation: SD, MD, MCN, SC, CB, MR, AD.

Formal analysis: SD, MR, NW, XNS, MC.

Data curation: SD, NW.

Interpretation: SD, MR, AD, MD, TS, XNS, MC.

Drafting the manuscript: MR, SD, TS.

Reviewing and editing the manuscript: all authors.

Supervision: MR.

Project administration: MCN, TS, MR.

## Consent for publication

Not applicable.

## Ethics approval and consent to participate

The study was conducted in accordance with the Declaration of Helsinki and was approved by the appropriate ethical and regulatory body (*Comité de Protection des Personnes Île-de-France* VI). All participants or their next of kin provided written informed consent.

## Funding

This work was supported by a grant "Legs Poix" from "Chancellerie de l’Université de Paris", Paris, France, the EMMA-0030 grant from the French "Agence Nationale pour la Recherche", and an unrestricted research grant from Air Liquide Medical Systems Inc., France. The research association "Association pour le Développement et l’Organisation de la Recherche en Pneumologie et sur le Sommeil" (ADOREPS) participated in the study funding and administrative support. None of the funding sources intervened in study design, data acquisition and management, or the writing of the manuscript.

## Availability of data

The complete data set used in this study will be made available to researchers upon reasonable request addressed to the corresponding author.

## Declaration of competing interest

SD, XNS, MCN, NW, MD, CB, SC, MC, AD have nothing to disclose regarding the present study or outside it.

TS has nothing to disclose regarding the present study. Outside this study, he reports, over the last 3 years, personal fees for consulting and teaching activities from Chiesi France, OSO-AI, and Lowenstein Medical France. He is a stock shareholder of startup Austral Dx. He is listed as inventor on several issued or pending patents in the field of respiratory physiology, respiratory neurophysiology, and respiratory care.

MR has nothing to disclose regarding the present study. He is listed as inventor on two issued or pending patents in the field of respiratory neurophysiology
