# Supplementary material for: Opioid-associated modulation of respiratory-related cortical activity in dyspnoeic mechanically ventilated patients: An electroencephalographic study
Source: Ann Intensive Care. 2026 Jan 16;16:100004. doi: 10.1016/j.aicoj.2025.100004 (PMC12934421; doi:10.1016/j.aicoj.2025.100004)
Supplement: Supplementary file 1 [file mmc1.docx]

**Electronic supplement to**

Opioid-associated modulation of respiratory-related cortical activity in dyspneic mechanically ventilated patients: an EEG study

**by** *Suela DEMIRI et al.*

**Table S1.** Respiratory Distress Observation Scale

*(Campbell ML, Templin T, Walch J. A Respiratory Distress Observation Scale for patients unable to self-report dyspnea. J Palliat Med 2010: 13: 285-290)*

| Variables | 0 | 1 | 2 |
| --- | --- | --- | --- |
| Heart rate min^_1^ | < 90 | 90-109 | ≥ 110 |
| Respiratory rate min^_1^ | <19 | 19-29 | ≥ 30 |
| Restlessness: non-purposeful movements | none | slight rise | pronounced rise |
| Paradox abdominal respiration | none |  | present |
| Respiratory accessory muscle activation | none | mild | intense |
| Grunting at end-expiration | none |  | present |
| Nasal flaring | none |  | present |
| Look of fear | none |  | present |

The RDOS score is the sum of the scores for each of its components.

**Figure S1**. Processing sequence used for continous covariance-based EEG connectivity analysis


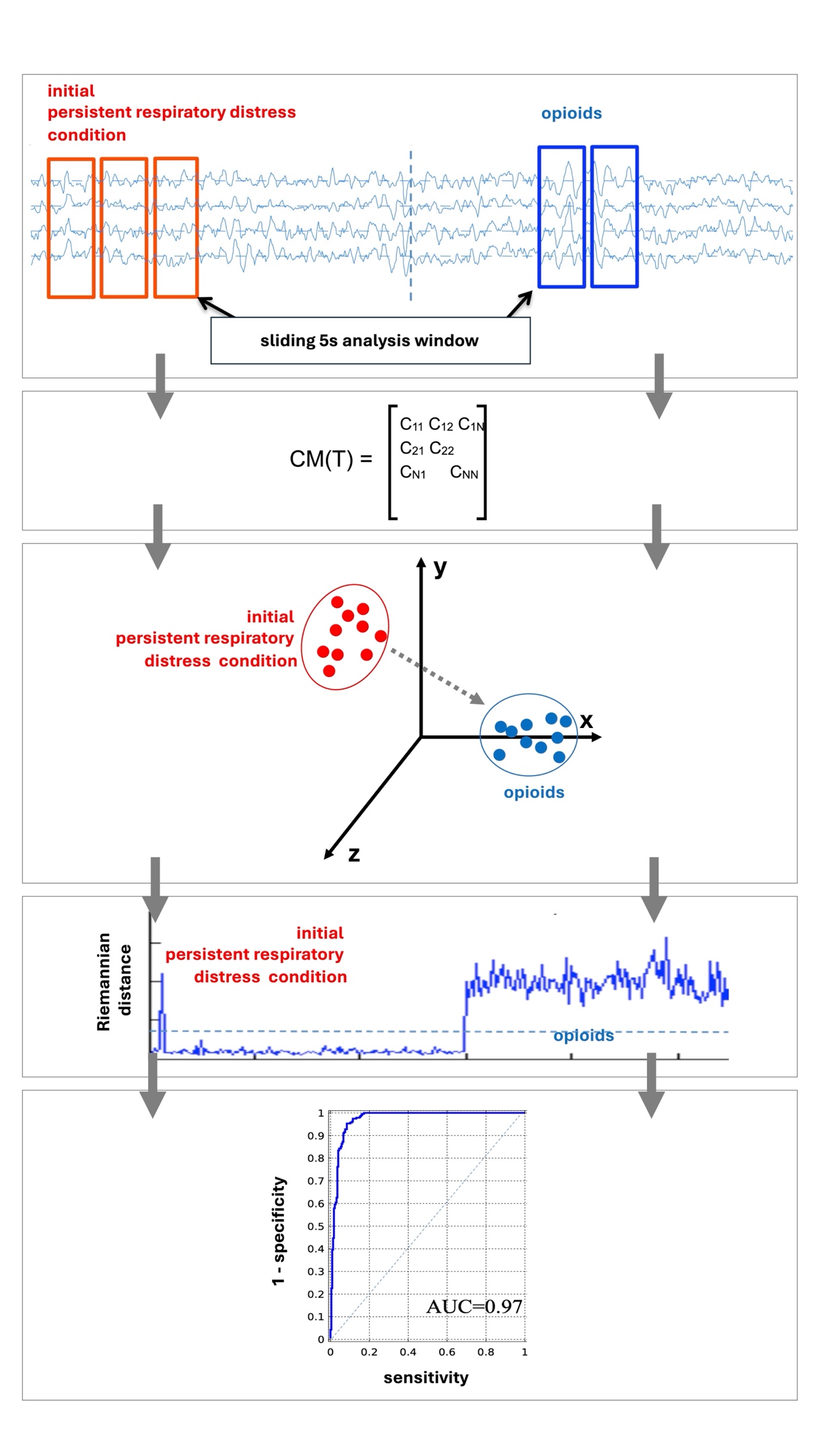


EEG data were segmented into overlapping 5-second sliding windows (first box). In the Riemannian geometry framework, each window was represented by a point derived from its covariance matrix (second box). The collection of these points formed a 'cloud' (third box) for each condition, allowing the comparison of distances between conditions (fourth box). The optimal Riemannian distance for distinguishing between conditions was determined by analyzing the area under the curve (AUC) in a sensitivity-specificity study (fifth box)
